# Food Safety Knowledge, Attitudes, and Practices of Brazilian Food Truck Food Handlers

**DOI:** 10.3390/nu11081784

**Published:** 2019-08-02

**Authors:** Lígia Isoni Auad, Verônica Cortez Ginani, Elke Stedefeldt, Eduardo Yoshio Nakano, Aline Costa Santos Nunes, Renata Puppin Zandonadi

**Affiliations:** 1 Department of Nutrition, Faculty of Health Sciences, University of Brasilia (UnB), Campus Darcy Ribeiro, Asa Norte, Brasilia 70910-900, Brazil; 2 GeQual—Study Group of Food Quality, Centro de Desenvolvimento do Ensino Superior em Saúde, Universidade Federal de São Paulo, São Paulo 04021-001, Brazil; 3 Department of Statistics, Institute of Exact Sciences, University of Brasilia (UnB), Campus Darcy Ribeiro, Asa Norte, Brasilia 70910-900, Brazil; 4 Department of Pharmacy, Faculty of Health Sciences, University of Brasília (UnB), Campus Darcy Ribeiro, Asa Norte, Brasilia 70910-900, Brazil

**Keywords:** food truck, food handlers, KAP, microbiological assessment, food safety

## Abstract

This study aimed to (i) compare the food safety knowledge, attitudes, and self-reported practices (KAP) and observed food safety practices of food truck (FT) food handlers, (ii) evaluate the microbiological quality of food and water samples collected from these vehicles, and (iii) establish a score classification for the KAP instrument according to the food contamination probability assessment. This study was conducted in three stages with 40 food truck food handlers conveniently sampled in the Federal District, Brazil, through structured interviews, application of an observational checklist for the assessment of handlers’ practices and the collection of food and water samples for determination of microbiological quality. FTs that are likely to exhibit food contamination and are at a high risk of foodborne diseases if at least one of the following situations occur: (1) if a food handler scores ≤6 in the knowledge section; (2) if a food handler scores ≤5 in the attitudes section; or (3) if a food handler scores ≤6 in the self-reported practices section. On the other hand, FTs in which handlers score higher than the cutoff points in all the sections are unlikely to exhibit food contamination and are at a low risk of foodborne diseases. The findings of this study are the first step to understand food handlers’ point of view and the initial diagnosis to guide educational strategies in the FT sector.

## 1. Introduction

Over the past decades, global eating patterns have been changing due to the process of urbanization and globalization [1]. Eating out of home is booming due to the difficulty to prepare food and the lack of time for consumption, along with the increasing demand for food diversification, availability, and accessibility [2]. The most recent data from the Brazilian household budget survey [3] showed that food purchase spent with food outside of the home increased 30% growth in six years in Brazil (from 24.1% in 2002–2003 to 31.1% in 2008–2009) [3].

Following the increase of the outside home food consumption, the emerging industry of food trucks (FTs) is facing rapid expansion, representing one of the best performing segments in the food service. The US FT industry was valued at US$ 856.7 million in 2015, with a forecast for 2020 of US$ 996.2 million [4]. In Brazil, the annual revenue of FTs in 2014 was 140 billion reais (about 35 billion USD) [5]. This industry is expected to continue thriving since FTs positively impacts the local and national economies. For owners and vendors, the FT activity offers economic and logistic advantages when compared to brick and mortar restaurants, besides representing a source of income, employment, and opportunity to start up their own business [6]. Moreover, beyond offering accessibility and convenience, FTs have become a powerful consumer trend due to their hedonic and social values—they offer affordable and high-quality dining options and represent a moment of leisure and celebration at which consumers can establish group bonds [7].

Given its importance and significant role in the food consumption industry outside of home, FTs need to ensure access to safe food considering the hygienic–sanitary perspective, since they are itinerant commercial kitchens that must operate in compliance with the local and national sanitation requirements. Also, FTs are, at the same time, similar to brick and mortar restaurants—regarding the special attention needed to time and temperature control during the food process chain—and the street food vending sector—when considering their selling points and exposure to the environmental conditions [6]. Our previous work performed in Brazil [8] revealed that FTs had a high level of inadequacy regarding their hygienic–sanitary practices and conditions, as well as a high rate of contaminated food samples, which raises the risk of foodborne diseases (FBDs) outbreaks.

FBDs are the result of the ingestion of water or food contaminated with microorganisms or toxins, typically leading to clinical severe symptoms and, in critical cases, death. Although preventable, FBDs remain a major public health challenge and an important contributor to morbidity and mortality worldwide, despite the industries and governments’ effort to ensure the hygienic–sanitary quality of food production. According to the World Health Organization (WHO) [9], in 2010 approximately 600 million cases of diseases and 420,000 deaths occurred by 31 foodborne hazards, including bacteria, viruses, parasites, toxins, and chemicals. WHO also states that in developing countries—including Brazil—there is a higher risk of the occurrence of FBDs, which directly impacts their society’s health and economic development [9].

The factors that can contribute to FBD outbreaks in some countries, including Brazil, are aspects related to time and temperature; contamination by food handlers, equipment, and utensils; contaminated raw material and water; and indirect contamination [10,11]. Even though food contamination can occur at any point of the production chain, food handling personnel play an essential role in ensuring food safety throughout the food production and storage chain by adhering to good personal hygiene and hygienic food handling practices. Food handlers are defined as individuals who are fully or partially involved in the food preparation process that come into contact with food and food contact surfaces, including those who harvest, slaughter, storage, transport, process, and prepare food [12,13]. Therefore, they can be vectors for food contamination directly, by leading to the transmissions of pathogens to food, and indirectly, by favoring cross-contamination [14]. Improper food handling by handlers has been responsible for foodborne outbreaks worldwide [15] and is commonly reported as a contamination-contributing factor [16] to foodborne outbreaks.

The enforcement of effective strategies to evaluate the food production and prevent contamination is essential to control the production process and provide safe food [17]. From this perspective, it is crucial to build and establish a culture of food safety in which food handlers produce and safely provide food, as well as know the associated risks with food handling and how to manage them [18]. Therefore, handlers’ awareness of their critical role and responsibility in food safety, as well as their knowledge and skills, are of crucial importance for handling food safely [19]. However, previous studies have reported a deficit of knowledge and improper food handling practices among food handlers in food services, including restaurants, hospitals, street food, universities and hotels [20,21,22,23,24].

Ensuring food safety of FTs is a major challenge. These vehicles are exposed to environmental conditions and usually do not provide adequate infrastructure to ensure the safe production of food. Also, a large number of meals are readily served to a variety of populations—including vulnerable groups such as children, pregnant women, the immune-compromised, the elderly and others at a higher risk of FBDs. Also, due to their itinerant and temporary nature, FTs generally escape effective food safety regulation and inspection [8], which potentially encourage the hiring of less qualified/training staff and, thus, increases the risk of FBDs. Given the growing presence of FTs in urban settings and the lack of information concerning the level of knowledge, attitudes, and self-reported practices (KAP) among their food handlers in Brazil, it is fundamental to understand the FT scenario by determining the risk associated with these vehicles and identifying their major deficiencies in order to formulate strategies and develop policies that can contribute to the safety of food and the decrease of the risk of FBDs. Therefore, this study aimed to compare the food safety knowledge, attitudes, and self-reported practices and observed food safety practices of food truck food handlers, to evaluate the microbiological quality of food and water samples collected from these vehicles, and to establish a score classification for the KAP instrument according to the food contamination probability assessment.

## 2. Materials and Methods

This cross-sectional, quantitative and exploratory study was carried out in the Federal District (Brazil). We used a convenient sample of 40 FTs (34%) among the 118 registered in the Brazilian Federal District health surveillance. A convenience sample was used, since the local and frequency of FT activity is not standardized or previously informed. The exclusion criterion was the FT owner being unwilling to participate in the research.

This study was approved by the ethics commission of the University of Brasília (UnB) (No. 2.178.214). The written informed consent of all participants was obtained and we assured their anonymity and confidentiality throughout the study.

**Figure 1:**
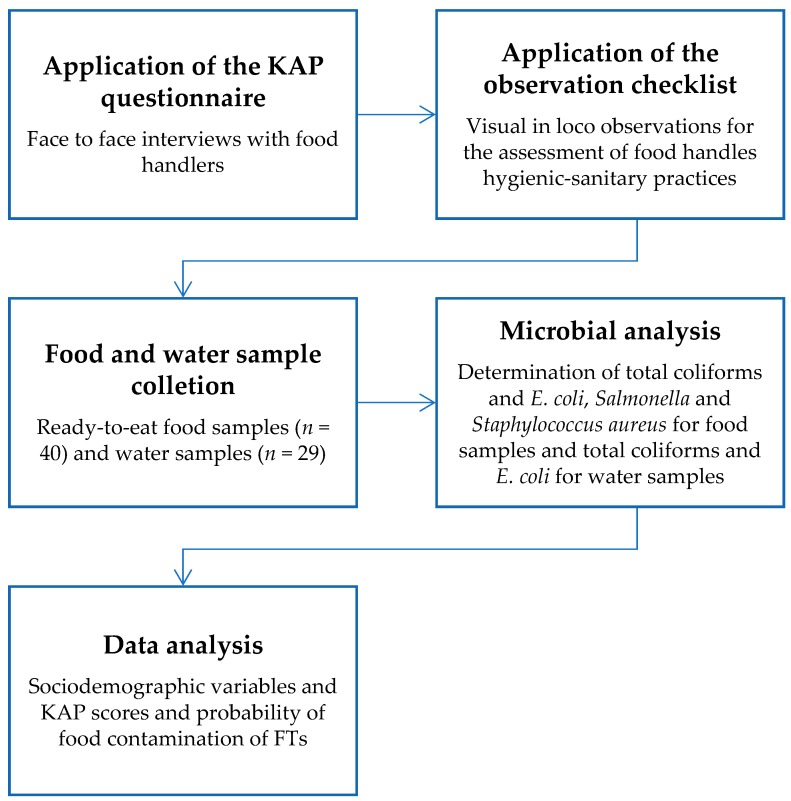
Layout of the study methodology.

This study was carried out according to the stages presented in Figure 1, which shows the schematic methodology outline.

### 2.1. Stage 1—Application of Knowledge, Attitudes and Self-Reported Practices (KAP) Questionnaire

A pilot study was performed with five FT food handlers. The data from the pilot study were not included in further analysis of the study. The KAP questionnaire used was adapted from previously published studies [22,25] and the WHO Five Keys to Safer Food [26]. After confirming that all the protocols elaborated for the research met their need, the application of the KAP questionnaire began.

We used a structured multiple-response questionnaire consisting of four sections to collect the demographic information of food handlers and to assess their level of KAP, which can be found in the Supplementary Materials. During the study period, FTs were selling different menu options in specific locations in clusters of between 6 to 10 vehicles. Forty (*n* = 40) food handlers agreed to participate in the face to face interview. Food handlers were unaware of the date of the visit and the application of questionnaires was carried out during the FT shift operation. All questionnaires were in Brazilian Portuguese and took 25–30 min to complete.

Section one was designed to obtain food handlers’ demographic information and consisted of gender, age, marital status, level of education, monthly income, experience in food service, and food safety training. Sections two, three, and four consisted of 10 multiple-choice questions each, and aimed to assess food handlers’ KAP. Knowledge questions had three possible answers (1 = true; 2 = false; and 3 = do not know/do not remember); attitudes questions were scored using a three-point Likert-type scale (1 = disagree; 2 = do not know/do not remember; and 3 = agree); and self-reported practices questions were assessed using a five-point rating scale (1 = never; 2 = rarely; 3 = sometimes; 4 = most of the times; and 5 = always).

The KAP scores were used to classify the FTs as to their probability of food contamination. The instrument score was obtained by assigning one point to each correct answer for each section separately, and a null score was attributed to each incorrect answer, as well as to ‘do not know/do not remember’, ‘rarely’ ‘sometimes’, and ‘most of the times’ answers. Therefore, the KAP instrument scores range from 0 to 10 points for each section, meaning that the higher the KAP score, the lower the probability of food contamination and, consequently, the lower the risk of FBDs.

### 2.2. Stage 2—Application of the Observation Checklist

Following the application of the KAP questionnaire, visual in loco observations were performed to assess the hygienic–sanitary practices of FT food handlers using a specific section of a checklist especially designed for this purpose. The checklist construction methods and validation were described and discussed in our previous studies [8,17].

The food handlers’ section of the checklist contained a total of five items, which were used to assess handlers’ personal hygiene routine and attitudes concerning food handling activities. The observations were carried out while food handlers were doing their routine activities. In order not to influence their attitudes during the assessment, food handlers were not aware of the observations.

### 2.3. Stage 3—Food and Water Sample Collection and Microbial Analysis

**Figure 2:**
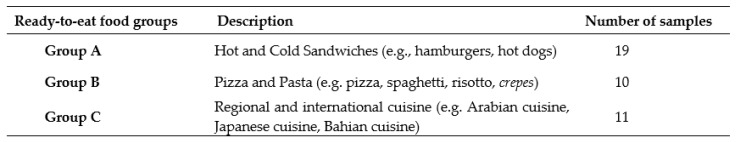
Classification of food trucks according to their ready-to-eat (RTE) food groups.

Following the interviews with food handlers and the application of the observation checklist, ready-to-eat (RTE) food samples (*n* = 40) were collected from each of the 40 FTs. For this study, FTs were divided into three groups according to their RTE foods, as shown in Figure 2. Water samples (*n* = 29) were obtained from 29 FTs–11 FTs (7 FTs from Group A, 2 FTs from Group B, and 2 from Group C) that had no water supply at the day of the collection. Selection criteria for the food sample selection included the most popular dish/product from the FT, according to data reported by the FT owner or manager.

All food samples were placed in a sterile plastic bag and immediately transported to the laboratory in containers with ice. Portions of each food sample weighing 25 g were diluted in 225 mL of 0.1% peptone water (Oxoid, UK). Food samples were homogenized in Stomacher (10^−1^ dilution) for 5 min at room temperature and then submitted to decimal serial dilutions to perform the microbiological analysis. Petrifilm™ plates (3M, St. Paul, MN, USA) were used to determine total coliforms (TC) and E. coli (EC), Salmonella (SL), and Staphylococcus aureus (SA), according to the manufacturer’s instructions. These bacteria were chosen because they are well-known indicators of Gram-negative and Gram-positive bacteria and potential-pathogenic microorganism markers. All analyses were performed in triplicate and on the same day of collection.

Considering the microbiological criteria recommended by the International Commission on Microbiological Specifications for Foods [27], food samples were considered microbiologically acceptable if coliforms counts were less than 10^2^ per g or ml, *E. coli* counts were less than 10^2^ CFU/g, while *Staphylococcus aureus* counts between 10^2^ and less than 10^3^ CFU/g and absence of *Salmonella* were considered acceptable. These parameters were used to evaluate the food samples and to classify the FTs into contaminated and not contaminated.

Water samples were collected from sinks of 29 FTs according to the Standard Methods of the American Public Health Association (APHA) [28]. Samples were collected into pre-sterilized 250 mL glass bottles which contained sodium thiosulfate (final concentration, 100 mg/liter) as chlorine neutralizer. Water samples were promptly placed in containers with ice and transported to the laboratory and were examined on the same day for the presence of coliforms and *E. coli* using Colilert (IDEXX, Chalfont St Peter, U.K.).

A sample of 100 mL was poured into a sterile bottle and the Colilert reagent was added. All Colilert samples were incubated at 37 °C and were examined after 24 h incubation. All samples which were yellow after incubation were examined under long wave ultraviolet light for a characteristic blue fluorescence, indicating the presence of *E. coli*. Considering the world health organization (WHO) guideline values for bacteriological quality of water [29], total coliform bacteria, and *E. coli* must not be detectable in any 100 mL sample. These parameters were used to evaluate water samples.

### 2.4. Stage 4—Data Analysis

Descriptive data were analyzed using the means and standard deviations for the quantitative variables and the frequencies and percentages for the categorical variables. All hypothesis tests were bicaudal and a *p*-value ≤0.05 was considered statistically significant. The statistical analysis considered the score of the three sections of the KAP instrument separately and the food samples collected.

The statistical analyses were performed using the IBM SPSS version 22.0 software (SPSS Inc., Chicago, IL, USA). Comparison of the KAP scores according to the sociodemographic variables assessed were performed as follows. The KAP scores were used to establish the probability of food contamination of FTs. A cutoff point was proposed for the classification of contamination probabilities of FTs, which was defined by the levels of sensitivity and specificity (area under the Receiver Operating Characteristic (ROC) curve (AUC)). A possible difference between the scores of the instrument among the variables considered was performed using the Mann–Whitney test for categorical variables with two groups and Kruskal–Wallis test with Müller–Dunn posthoc test for variables with three or more categories. The frequency of contamination was compared between groups of FTs using the Pearson Chi-square test.

## 3. Results

Table 1 summarizes the demographic characteristics of FT food handlers, the sample distribution according to the sociodemographic characteristics, and the association of those variables with the KAP scores.

The sociodemographic data showed that most of the food handlers were young (average age 32.6 ± 9.5 years, ranging from 22 to 55 years) males (80%), who were single, divorced, or widowed (55%) and had children (57.5%). The results also revealed that 42.5% and 37% had completed secondary and tertiary school, respectively, and 55% had undergone food safety training. Also, most food handlers (67.5%) reported having previous experience in the food service business. Regarding the monthly average income, almost 43% of the food handlers earned between 1 to 2 Brazilian minimum wages (US$ 249.5–499). Statistically significant differences can be observed in the attitudes scores regarding marital status (*p* = 0.029), monthly income (*p* = 0.018), and food safety training (*p* = 0.033).

The mean score and standard deviation of the KAP instrument according to food truck classification and samples contamination status are presented in Table 2. We divided FT classification into three groups, and food and water contamination status category into not contaminated and contaminated, according to the criteria previously mentioned.

Significant differences in the KAP scores can be observed in the knowledge section, concerning food contamination status, with contaminated FTs obtaining a lower score than not contaminated. Statistically significant differences were also found in the attitudes section regarding FT classification. The attitudes score of Group A was significantly lower when compared to Groups B and C scores (*p* = 0.013), but they did not significantly differ between Groups B and C.

Table 3, Table 4 and Table 5 show the results of the food safety knowledge, attitudes, and self-reported practices assessment based on the KAP questionnaire. The correct answers are indicated in bold.

Table 3 shows that question 1, which relates to hand washing, had the highest success rate. Parallelly, at least 80% of handlers correctly answered the knowledge questions 2, 4, 5, 6, and 8. On the other hand, in questions 7 and 9, the success rate was less than 50%. More than two-thirds of handlers believed that contaminated food would always present changes in sensory aspects, and half of them believed that adequately cooked food is free from pathogenic bacteria.

Table 4 describes questions related to food safety attitudes. Question 8 had the highest success rate, demonstrating handlers’ awareness of the effects of improper food storage. Food handlers obtained a success rate of at least 95% in 50% of the ten questions. Additionally, questions 5 and 7 obtained the lowest success rate in this section. Only 1 out of 5 handlers correctly answered that eggs do not need to be washed after purchase, and only 7.5% correctly agreed that storing hot food in the fridge is not a problem.

Table 5 shows that 7 out of 10 self-reported practice questions obtained at least 70% of success rate. On the other hand, questions 3 and 6, which presented the lowest success rates, demonstrate that 65% of handlers perform incorrect food thawing procedures and 60% talk while handling ready-to-eat food.

Table 6 lists the items assessed during the observations carried out with FT food handlers. The assessment of these practices was performed without respondents’ awareness and during their working shift, in order to maintain handlers’ natural behavior. Among the observed food safety practices, the item concerning the use of uniform exclusively for work and food handling obtained the highest inadequacy percentage rate. In the FTs assessed, handlers usually move to work by bus or motorcycle, meaning that they are regularly exposed to air pollution and dirt. Items concerning the use of caps and adornments and the performance of unhygienic acts while handling food also displayed a high inadequacy percentage rate. Handlers obtained a moderate inadequacy percentage rate concerning the practice of washing hands. On the other hand, a low inadequacy percentage rate was observed for the exclusion of the handler in cases of illness or infections. The results of these observations were later compared to the results handlers obtained from the KAP assessment in order to determine the degree of agreement between them.

**Figure 3:**
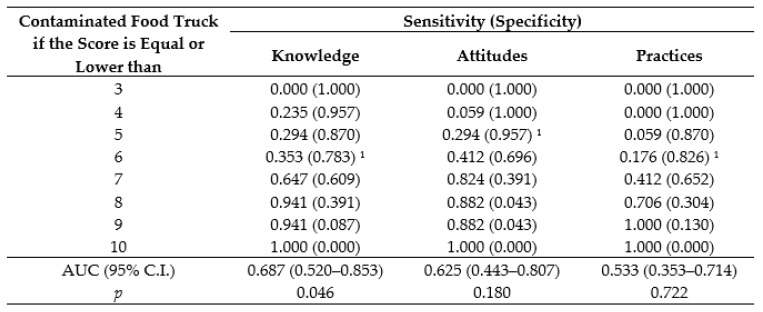
Probability of contamination of food truck defined by sensitivity and specificity levels according to the cutoff point adopted for the sections of the KAP instrument. ^1^ Cutoff point which sets the minimum specificity of 70%; AUC: Area under the Receiver Operating Characteristic (ROC) curve.

The KAP scores of each section were used to establish the probability of food contamination of FTs, which was defined by the levels of sensitivity and specificity. Considering the contamination as the outcome of interest, sensitivity represents the probability of the KAP instrument to present a positive result, i.e., a handler obtains a low score in one or more sections of the KAP assessment in contaminated FTs, while specificity represents the probability of the KAP instrument to present a negative result, i.e., a handler obtains a high score in all sections of the KAP instrument in noncontaminated FTs. As shown in Figure 3, the cutoff points 6, 5, and 6 set the minimum specificity of 70% for the knowledge, attitudes, and self-reported practices sections, respectively.

**Figure 4:**
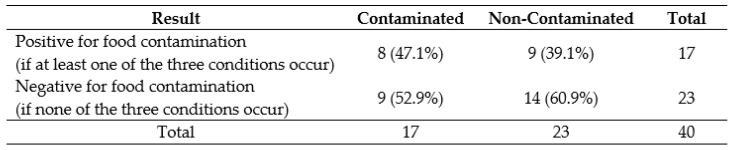
Classification of food trucks considering the cutoff points.

Based on these results, a FT is likely to exhibit food contamination—and, therefore, is at high risk of FBDs—if at least one of the following situations occur: (1) if a food handler scores ≤6 in the knowledge section; (2) if a food handler scores ≤5 in the attitudes section; or (3) if a food handler scores ≤6 in the self-reported practices section. On the other hand, FTs in which food handlers score higher than the cutoff points set in all sections are unlikely to exhibit food contamination and are at low risk of FBDs. This classification rule resulted in a sensitivity of 47.1% and a specificity of 60.9% and correctly classified 55.0% of the FTs, as shown in Figure 4. It also correctly classified 63.2% of the FTs from Group A, 40.0% of the Group B, and 54.5% of Group C.

## 4. Discussion

The profile of FT food handlers observed in this study is quite diverse from those reported for food handlers in the scientific literature. Despite the same findings concerning age and gender of food handlers having been observed in Malaysia [30], Lebanon [31], Jordan [21], and Romania [32], a higher proportion of young female food handlers is usually observed in studies [20,23,25,33,34,35,36,37,38]. Similarly, the educational background of the respondents in this study differs from numerous surveys [25,30,33,36,37,39,40], which commonly report low levels of trained and educated food handlers.

Women are more likely to be involved in food handling activities since they are traditionally responsible for and skilled in food handling and preparation, as well as providing food for households [41]. Parallelly, food handlers with poor education and training levels are commonly reported in food services around the world, especially in the street food sector of developing countries, where this informal business represents a source of employment and income for unskilled workers [42]. However, in our study, we observed a higher proportion of young male handlers, with high levels of training and educational attainment. From the 40 food handlers interviewed, nine (22.5%) declared to have a family relationship with the owner, while 12 (30%) were at the same time the owners and food handlers of the FTs (data not shown). Results of our previous work [6] show that owners started a FT business mainly due to unemployment and the opportunity of having their own business. Besides, the presence of family members can be considered a survival strategy, since unpaid family labor allows higher incomes to vendors that otherwise would not be possible if they had done the same work alone or utilized paid labor. Another potential explanation for the presence of highly educated young males as FT food handlers may be the high unemployment rate among young people and heads of the household in Brazil [43]. Since there is a greater demand for highly qualified and experienced professionals, undergraduate and recently graduated professionals, as well as heads of family, face difficulties entering or reentering the labor market and, consequently, undertake less qualified jobs, becoming underemployed workers.

The results of the survey show significant differences in the attitudes scores regarding marital status (*p* = 0.029), monthly income (*p* = 0.018), and food safety training (*p* = 0.033). Respondents with companions, higher monthly incomes, and training had significantly higher attitudes scores. There is no study in the literature associating marital status or monthly incomes with higher attitudes scores. However, in a study conducted with food service staff in Jordan, Osaili et al. [21] found that respondents with higher salaries had significantly better food safety knowledge than those with lower salaries. In our study, despite not statistically significant, handlers with higher incomes also had higher knowledge scores than those with lower incomes. As to food safety training, it was expected that trained handlers would achieve significantly higher scores than untrained handlers, since food handlers with training in food safety are more likely to exhibit positive food safety behavior. Findings indicating a significant impact of food safety training on attitudes were also observed in by Nee and Sani [44] and Key Lee et at. [45] in Malaysia, and by Nyabera et al. [46] in Kenya.

Significant differences in the KAP scores can also be observed in the attitudes section regarding FT classification, and in the knowledge section concerning food contamination status. The attitudes score of Group A was significantly lower when compared to Groups B and C scores (*p* = 0.013), but they did not significantly differ between Groups B and C. Despite not being statistically significant, knowledge scores from Group A were also lower than those of Groups B and C. FTs with contaminated food samples also displayed significantly lower scores in the knowledge section (*p* = 0.042)—and, despite not being statistically significant, lower scores in attitudes and self-reported practices scores—when compared to FTs with no food contamination. These findings are consistent with our previous observations [8], in which FTs from Group A obtained the highest inadequacy percentage concerning the hygienic–sanitary conditions and practices evaluated and, therefore, the highest probability of food contamination when compared to Groups B and C. These differences among FT groups are directly related to their food preparation processes.

As previously mentioned, FTs were classified according to their RTE foods, in which FTs from Group A sell hot and cold sandwiches, FTs from Group B serve pizza and pasta, while FTs from Group C prepare regional and international dishes. Even though all FTs have a high number of ingredients and food preparation processes, FTs serving hot and cold sandwiches have a more complex food chain when compared to the other options. Brown et al. [47] have identified that the complexity of preparation processes is a potential contributing factor for the occurrence of FBDs outbreaks. As multi-ingredient dishes, sandwiches usually require more intensive handling during preparation and serving, which consequently increases opportunities for contamination. In fact, foods with multiple ingredients were identified most frequently with worldwide outbreaks, including in Brazil [15,48,49]. In addition, food handlers’ lack of knowledge and negligence in food handling are important factors associated with food contamination, which are also a possible explanation for the findings of our study.

No significant difference (*p*>0.05) occurred in the knowledge, attitudes, or self-reported practices scores for gender, age, level of education, length of FT service, or previous experience. In contrast to our findings, other studies have indicated a significant relationship among those variables and the KAP scores of food handlers. Bou-Mitri et al. [31] reported higher attitude and overall KAP scores for food handlers of Lebanese government hospitals with long working experience. Parallelly, Sharif et al. [50] found higher overall KAP scores for females and handlers with higher education levels and Norhaslinda et al. [51] for older food handlers.

Considering all FT food handlers assessed (*n* = 40), in the knowledge section, the mean score of handlers was 7.23 ± 1.78, with a minimum score of 4 (40%) and a maximum of 10 (100%). In the attitudes section, handlers obtained a mean score of 6.85 ± 1.21, with a minimum score of 4 (40%) and a maximum of 9 (90%). In turn, the mean score of handlers in the self-reported practices section was 7.73 ± 1.38, with a minimum score of 5 (50%) and a maximum score of 10 (100%). Although these results might seem to imply a good level of knowledge, attitudes, and self-reported practices of food handlers, some important questions had a very low rate of right answers. In questions concerning food contamination and FBDs (Table 3, knowledge section, questions 7 and 9), food temperature (Table 4, attitudes section, questions 5, 6, and 7, and Table 5, self-reported practices section, question 3) and attitudes while handling food (Table 4, attitudes section, question 6), handlers achieved scores lower than 50%.

In addition, it is important to highlight that handlers’ knowledge, attitudes, and self-reported practices are inconsistent with the observations carried out. Although all food handlers correctly answered that washing hands before work reduces the risk of food contamination and almost all (95%) claimed that they washed hands before handling food, the observations show that only 50% performed handwashing. Almost all handlers (95%) were also aware that the use of adornments, accessories, or jewelry can contaminate food and that wearing a cap can reduce food contamination. Moreover, when handling food, 77.5% of handlers reported never using jewelry or adornments, and 82.5% claimed to keep their hair completely covered with a cap. Nonetheless, the observations revealed that only 17.5% of the handlers adequately protected their hair with a cap and wore no adornments while performing food handling activities. Handlers’ knowledge, attitudes, and self-reported practices are equally conflicting regarding acts such as talking, singing, and handling money during food handling operations.

The low agreement between knowledge, attitudes, and self-reported practices and the observations are not surprising. Even though theoretical training based on KAP is a commonly used technique to improve handlers’ food safety performance, this method of providing information has serious limitations. One of the training’s major flaws is the assumption that the received information is translated into practices and behavior. An integrative review conducted by Zanin et al. [52] on the association of food handlers KAP with training in food safety revealed that 50% of the selected studies reported that knowledge was not translated into attitudes or practices change, which concurs with the findings of our study. The occurrence of a social desirability bias may also have contributed to the divergence between self-reported and observed practices of handlers. Social desirability bias is described as the tendency of respondents to give socially desirable responses in such a way as to be viewed favorably by others [53]. This tendency can be expressed by overreporting and overestimating a socially desirable behavior. As previously mentioned, handlers were aware of the hygiene practices and the importance of complying with them, but they were not willing to admit their noncompliance.

Considering the scores obtained in the KAP instrument application, cutoff points for each section were proposed for the classification of food contamination probabilities of FTs. The adopted cutoff points correctly classified 55.0% of the contaminated FTs (sensitivity of 47.1% and specificity of 60.9%). We observed similar results when FTs were classified according to their RTE food groups: the cutoff point adopted correctly classified 63.2% of FTs from Group A (hot and cold sandwiches); 40.0% of FTs from Group B (pizza and pasta); and 54.5% of FTs from Group C (regional and international). The adoption of the KAP instrument as a diagnostic strategy and a complementary tool to the evaluation instrument validated in our previous studies [8,17] can effectively assist in the decision-making process and the development of targeted interventions strategies for the prevention and control of FBDs. It is worth noting that the KAP instrument assessment described in this study is considered applicable to other contexts if all the questions in the knowledge, attitudes, and self-reported practices sections remain unchanged.

It is important to emphasize that as worrying as food contamination is water contamination, which has emerged as a major concern regarding food safety in this study. From the 40 FTs assessed, 11 had no water supply to perform hygiene practice—such as hand hygiene and other sanitation needs—and, from the 29 FTs with water supply, 13 (44.8%) presented bacterial contamination. Contaminated water may be a potential source of food contamination. In fact, data from the Brazilian Ministry of Health show that water was the most implicated source in FBDs outbreaks notified in Brazil in 2018 [49]. Therefore, this finding has emerged as an important public health concern and needs to be a subject of further studies.

## 5. Conclusions

This study has some potential limitations. Due to its small sample size and convenient nature, the data presented here may not be representative of the FT scenario of the Brazilian Federal District or other regions worldwide. Throughout this study, 63 potentially eligible FTs were located and invited to participate in the interview. However, we obtained a high rejection rate (32%, 20 FTs) regardless of the use of a written-consent approach and the awareness of the anonymity and confidentiality of the study by the participants. Repeated attempts, aiming to increase the sample of FT food handlers, were unsuccessful. A second limitation is the conduction of face to face interviews, considering food handlers’ attitudes and self-reported practices, which could have contributed to the occurrence of a social desirability bias. Future research should consider the assessment of the social desirability bias, as well as other interview methods which eliminate interviewer effects, in order to minimize the degree of this tendency. Additionally, further investigations should involve the conduction of interviews with FT food handlers in other sites to identify possible generalizations and to comprehend the potential regional differences. Also, since this is a cross-sectional survey, we recommend longitudinal studies to obtain more in-depth FT food handlers’ information, such as the social and psychological factors driving their attitudes and practices to guide education and training programs.

Despite these limitations, this study has considerable potential for the food sector. Since food handlers are a ubiquitous figure in the food market worldwide and are the main responsible for FBDs outbreaks, identifying the potential gaps in their knowledge, attitudes, and self-reported practices is imperative to building upon interventional strategies to promote food safety and prevent the occurrence of FBDs. The ease of application and score interpretation of the KAP instrument, as well as its cost-effectiveness, suggest it may assist in recognizing the potential weaknesses in the KAP triad of food handlers. Additionally, the recognition of those deficiencies may provide a new approach for the strategic planning and execution of training programs, from the academic and practical perspectives, aiming at effective food safety management. For food handlers, it means to provide the knowledge necessary to properly evaluate and decide on safe food practices. It is also fundamental to establish an organizational culture which positively influences individuals, since environmental factors are key determinants of motivation to transfer training to workplace and to behavior change.

The findings of this research provide essential information concerning food handlers’ level of knowledge, attitudes, and self-reported practices, as well as the contamination status of water and food samples. FT food handlers had an unsatisfactory level of knowledge, attitudes, and self-reported practices on essential concepts of food safety. In addition, attitudes and self-reported practices had a low agreement with the observations carried out. The high contamination rate of food and water samples were another serious concern of this study. These results indicate a potential increase in the probability of food contamination and, in turn, in the risk of FBDs outbreaks in the FT sector. Nonetheless, these results, along with the KAP instrument scoring system, are the first step to understand food handlers’ point of view and the initial diagnosis to guide educational strategies aiming the promotion of a food safety culture in FTs in Brazil and potentially in other countries.

## Figures and Tables

**Table 1:** Sociodemographic variables and their association with knowledge, attitudes and self-reported practices (KAP) scores of food truck food handlers studied in the Federal District, Brazil (*n* = 40).

Variables (*n*; %)	Knowledge		Attitudes		Practices	
	Mean (SD)	*p*-Value	Mean (SD)	*p*-Value *	Mean (SD)	*p*-Value *
**Total**	7.23 (1.78)		6.85 (1.21)		7.73 (1.38)	
**Gender**						
Male (32; 80.0%)	6.97 (1.75) ^A^	0.068	6.75 (1.29) ^A^	0.271	7.84 (1.25) ^A^	0.375
Female (8; 20.0%)	8.25 (1.58) ^A^		7.25 (0.71) ^A^		7.25 (1.83) ^A^	
**Age**						
≤25 (12; 30.0%)	6.92 (2.02) ^A^		6.25 (1.14) ^A^		7.67 (1.61) ^A^	
26–40 (22; 55.0%)	7.14 (1.61) ^A^	0.282	7.00 (1.23) ^A^	0.097	7.95 (1.21) ^A^	0.345
>40 (6; 15.0%)	8.17 (1.84) ^A^		7.50 (0.84) ^A^		7.00 (1.41) ^A^	
**Marital Status**						
With companion (18; 45.0%)	7.50 (1.76) ^A^	0.287	7.33 (1.14) ^A^	0.029	7.83 (1.46) ^A^	0.634
Without companion (22; 55.0%)	7.00 (1.80) ^A^		6.45 (1.14) ^B^		7.64 (1.33) ^A^	
**Children**						
No (17; 42.5%)	7.24 (1.89) ^A^	0.967	7.12 (1.41) ^A^	0.123	7.82 (1.59) ^A^	0.527
Yes (23; 57.5%)	7.22 (1.73) ^A^		6.65 (1.03) ^A^		7.65 (1.23) ^A^	
**Level of Education**						
Primary (Elementary School) (8; 20.0%)	7.00 (1.41) ^A^		6.88 (0.64) ^A^		8.38 (0.92) ^A^	
Secondary (High School) (17; 42.5%)	7.24 (2.11) ^A^	0.843	6.41 (1.37) ^A^	0.111	7.47 (1.46) ^A^	0.368
Tertiary (Graduate) (15; 37.5%)	7.33 (1.63) ^A^		7.33 (1.11) ^A^		7.67 (1.45) ^A^	
**Monthly Income (minimum wage; US)**						
≤2 (≤ US $499) (22; 55.0%)	6.86 (1.91) ^A^	0.154	6.41 (1.18) ^A^	0.018	7.50 (1.26) ^A^	0.269
>2 (> US $499) (18; 45.0%)	7.67 (1.53) ^A^		7.39 (1.04) ^B^		8.00 (1.49) ^A^	
**Lenght of FT Service**						
≤1 year (17; 42.5%)	7.18 (2.01) ^A^	0.900	6.82 (1.24) ^A^	0.767	7.41 (1.42) ^A^	0.304
>1 year (23; 57.5%)	7.26 (1.63) ^A^		6.87 (1.22) ^A^		7.96 (1.33) ^A^	
**Previous Experience**						
No (13; 32.5%)	6.69 (2.02) ^A^	0.196	7.00 (1.29) ^A^	0.676	7.62 (1.56) ^A^	0.812
Yes (27; 67.5%)	7.48 (1.63) ^A^		6.78 (1.19) ^A^		7.78 (1.31) ^A^	
**Food Safety Training**						
No (18; 45.0%)	7.00 (2.14) ^A^	0.638	6.39 (1.29) ^A^	0.033	7.28 (1.41) ^A^	0.085
Yes (22; 55.0%)	7.41 (1.44) ^A^		7.23 (1.02) ^B^		8.09 (1.27) ^A^	

SD: Standard deviation; * Mann–Whitney test for categorical variables with two groups and Kruskal–Wallis test with Müller–Dunn posthoc test for variables with three or more categories; ^A, B^ Groups with the same letters do not differ significantly.

**Table 2:** Food truck classification and food and water samples contamination status and their association with KAP scores of food truck food handlers studied in the Federal District, Brazil (*n* = 40).

Variables (*n*; %).	Knowledge		Attitudes		Practices	
	Mean (SD)	*p*-Value *	Mean (SD)	*p*-Value **	Mean (SD)	*p*-Value **
**Total**	7.23 (1.78)		6.85 (1.21)		7.73 (1.38)	
**FT Classification**						
Group A (19; 47.5%)	6.74 (2.00) ^A^		6.26 (1.10) ^A^		7.79 (1.13) ^A^	
Group B (10; 25.0%)	8.00 (1.49) ^A^	0.291	7.50 (1.18) ^B^	0.013	7.20 (1.62) ^A^	0.446
Group C (11; 27.5%)	7.36 (1.43) ^A^		7.27 (1.01) ^B^		8.09 (1.51) ^A^	
**Food Contamination**						
Not contaminated (23; 57.5%)	7.70 (1.64) ^A^	0.042	7.09 (1.00) ^A^	0.166	7.78 (1.51) ^A^	0.714
Contaminated (17; 42.5%)	6.59 (1.80) ^B^		6.53 (1.42) ^A^		7.65 (1.22) ^A^	
Water Contamination *						
Not contaminated (16; 55.2%)	7.31 (1.93) ^A^	0.982	6.85 (0.99) ^A^	0.767	8.00 (1.53) ^A^	0.138
Contaminated (13; 44.8%)	7.38 (1.82) ^A^		6.88 (1.31) ^A^		7.19 (1.33) ^A^	

SD: Standard deviation; * 11 (27.5%) food trucks with no water samples; ** Mann–Whitney test for categorical variables with two groups and Kruskal–Wallis test with Müller–Dunn posthoc test for variables with three or more categories; ^A, B^ Groups with the same letters do not differ significantly.

**Table 3:** Food safety knowledge assessment of food truck food handlers studied in the Federal District, Brazil (*n* = 40).

Question	Number of Responses (%)		
	True	False	Do Not Know/Do Not Remember
1. Washing hands before work reduces the risk of food contamination.	40 (100%)	0 (0%)	0 (0%)
2. Wearing gloves is a substitute for hand cleansing.	6 (15%)	32 (80%)	2 (5%)
3. Freezing kills the microbes that may cause deterioration of foods and foodborne diseases.	13 (32.5%)	25 (62.5%)	2 (5%)
4. A healthy food handler may contaminate food with microbes that cause foodborne diseases.	32 (80%)	7 (17.5%)	1 (2.5%)
5. Food handlers’ health status must be periodically checked.	36 (90%)	3 (7.5%)	1 (2.5%)
6. Eating food one day past its expiration date poses risk to health.	35 (87.5%)	4 (10%)	1 (2.5%)
7. Food that is unfit for consumption always presents color, taste and/or smell changes.	27 (67.5%)	13 (32.5%)	0 (0%)
8. Washing fruit and vegetables under running water and peeling them is enough to make these foods safe for consumption.	6 (15%)	33 (82.5%)	1 (2.5%)
9. Well cooked food is free from microbes that cause foodborne diseases.	20 (50%)	17 (42.5%)	3 (7.5%)
10. Food handlers with cuts or wounds on hands do not need to be kept away from food handling activities.	14 (35%)	26 (65%)	0 (0%)

**Table 4:** Food safety attitudes assessment of food truck food handlers studied in the Federal District, Brazil (*n* = 40).

Question	Number of Responses (%)		
	Disagree	Do Not Know/Do Not Remember	Agree
1. Raw and cooked food should be stored separately.	2 (5%)	0 (0%)	38 (95%)
2. The use of adornments, accessories or jewelry can contaminate food.	1 (2.5%)	1 (2.5%)	38 (95%)
3. Wearing a cap is an important practice to reduce the risk of food contamination during handling.	1 (2.5%)	0 (0%)	39 (95%)
4. Defrosted food must not be refrozen.	10 (25%)	1 (2.5%)	29 (72.5%)
5. Eggs must be washed after purchase before being stored.	8 (20%)	8 (20%)	24 (60%)
6. Food thawing can be performed in a bowl with or without water in the sink at room temperature.	17 (42.5%)	1 (2.5%)	22 (55%)
7. Food must be cooled at room temperature before being put in the fridge.	3 (7.5%)	1 (2.5%)	36 (90%)
8. Improper food storage may pose risk to health.	0 (0%)	0 (0%)	40 (100%)
9. Preparing food in advance reduces the risk of contamination.	24 (60%)	3 (7.5%)	13 (32.5%)
10. Using non-sanitized fresh herbs in the decoration of a portion of broth or soup can contaminate these foods.	2 (5%)	0 (0%)	38 (95%)

**Table 5:** Food safety self-reported practices assessment of food truck food handlers studied in the Federal District, Brazil (*n* = 40).

Question	Number of Responses (%)				
	Never	Rarely	Sometimes	Most of the Times	Always
1. Do you wash your hands immediately before handling food?	0 (0%)	0 (0%)	1 (2.5%)	1 (2.5%)	38 (95%)
2. Do you use food after the expiration date if it has no change in quality aspect?	36 (90%)	1 (2.5%)	1 (2.5%)	1 (2.5%)	1 (2.5%)
3. Do you thaw food at room temperature (outside the fridge)?	14 (35%)	2 (5%)	6 (15%)	5 (12.5%)	13 (32.5%)
4. Do you check the expiration date of ingredients before using them in food preparation?	0 (0%)	0 (0%)	1 (2.5%)	4 (10%)	35 (87.5%)
5. Do you wash your hands after using the bathroom?	0 (0%)	0 (0%)	0 (0%)	0 (0%)	40 (100%)
6. Do you talk while handling ready to eat food?	16 (40%)	5 (12.5%)	9 (22.5%)	4 (10%)	6 (15%)
7. Do you handle food when you are sick or have cuts on hands?	26 (65%)	8 (20%)	6 (15%)	0 (0%)	0 (0%)
8. Do you wear nail polish or use jewelry when handling food?	31 (77.5%)	0 (0%)	2 (5%)	0 (0%)	7 (17.5%)
9. Do you keep your hair completely covered with a cap while handling food?	1 (2.5%)	1 (2.5%)	4 (10%)	1 (2.5%)	33 (82.5%)
10. Do you sanitize your workplace after finishing your service?	0 (0%)	0 (0%)	0 (0%)	0 (0%)	40 (100%)

**Table 6:** Items and frequency responses of the observations carried out with food truck food handlers studied in the Federal District, Brazil (*n* = 40).

Observed Food Safety Practice	Adequate (*n*, %)	Inadequate (*n*, %)
1. The personal hygiene routine of handlers includes the use of preserved and clean uniforms that are used exclusively during food handling operations, with protection against direct contact with food (coat or apron).	1 (2.5%)	39 (97.5%)
2. The personal hygiene routine of manipulators includes the use of hair trapped and protected with a cap and the use of a coat or apron. In case of a beard and mustache, a mask should be used. Nails are clean, short, with no enamel or base. No wearing of adornments (necklaces, amulets, bracelets, ribbons, earrings, nails, and false eyelashes, piercing on exposed areas, watches and rings) during food handling operations.	7 (17.5%)	33 (82.5%)
3. Handlers with cutaneous lesions and wounds or symptoms of diseases/infections (i.e., respiratory, gastrointestinal, ocular) are excluded from food handling operations.	30 (75.0%)	10 (25.0%)
4. Handlers do not smoke, sing, whistle, sneeze, spit, cough, eat, handle money or practice other acts that may contaminate food during food handling operations.	10 (25.0%)	30 (75.0%)
5. Handlers carefully wash their hands when they arrive at work, before and after handling the food, after any interruption of service, after touching contaminated materials, after using the toilets and whenever necessary. Under the impossibility of washing hands, handlers wear disposable gloves in place of utensils to handle only ready-to-eat foods and previously sanitized fruits and vegetables, replacing them and disposing of them as soon as they discontinue the procedure and before touching another food or surface that is not part of the preparation.	20 (50.0%)	20 (50.0%)

## Supplementary Materials

The following are available online at https://www.mdpi.com/2072-6643/11/8/1784/s1, File S1: Food truck food handler questionnaire.
