# Supplementary material for: Food Safety Knowledge, Attitudes, and Practices of Brazilian Food Truck Food Handlers
Source: Nutrients. 2019 Aug 2;11(8):1784. doi: 10.3390/nu11081784 (PMC6722497; doi:10.3390/nu11081784)
Supplement: Supplementary file 1 [file nutrients-11-01784-s001.pdf]

FOOD TRUCK FOOD HANDLER QUESTIONNAIRE

**Number:**	**Year:**
**Food Truck/Owner:**	
**Food Handler:**	
**Health License:**	
**Email/Phone:**	
**City/State:**	**Zip Code:**

SOCIO-DEMOGRAPHIC STATUS
**1. Gender**
1. Male<br>2. Female
**2. Age**
1. ≤25<br>2. 26-40<br>3. >40
**3. Marital status**
1. With Companion (Married or Stable Union)<br>2. Without Companion (Single/Divorced/Widowed)
**4. Children**
1. Yes<br>2. No
**5. Monthly income (minimum wage; US$)**
1. <1 (US$ 249.5)<br>2. 1-2 (US$ 249.5 - 499)<br>3. >2-4 (>US$ 499 – 998)<br>4. >4 (>US$ 998)
**6. Level of education**
1. Primary (Elementary School)<br>2. Secondary (High School)<br>3. Tertiary (Graduate)
**7. Previous Experience in Food Services**
1. Yes<br>2. No
**8. Are you related to the owner?**
1. Yes<br>2. No<br>3. I am the owner
**9. Food Safety Training**

1. Yes<br>2. No
**10. Have you received training in the last 6 months?**
1. Yes<br>2. No
**11. Do you have periodic medical examination?**
1. Yes<br>2. No
**KNOWLEDGE, ATTITUDES AND SELF-REPORTED PRACTICES QUESTIONNAIRE**
**KNOWLEDGE**
**1. Washing hands before work reduces the risk of food contamination.**
1. True<br>2. False<br>3. Don't know/Don't remember
**2. Wearing gloves is a substitute for hand cleansing.**
1. True<br>2. False<br>3. Don't know/Don't remember
**3. Freezing kills the microbes that may cause deterioration of foods and foodborne diseases.**
1. True<br>2. False<br>3. Don't know/Don't remember
**4. A healthy food handler may contaminate food with microbes that cause foodborne diseases.**
1. True<br>2. False<br>3. Don't know/Don't remember
**5. Food handlers' health status must be periodically checked.**
1. True<br>2. False<br>3. Don't know/Don't remember
**6. Eating food one day past its expiration date poses risk to health.**
1. True<br>2. False<br>3. Don't know/Don't remember
**7. Food that is unfit for consumption always presents color, taste and/or smell changes.**
1. True<br>2. False

3. Don’t know/Don’t remember
**8. Washing fruit and vegetables under running water and peeling them is enough to make these foods safe for consumption.**
1. True<br>2. False<br>3. Don’t know/Don’t remember
**9. Well cooked food is free from microbes that cause foodborne diseases.**
1. True<br>2. False<br>3. Don’t know/Don’t remember
**10. Food handlers with cuts or wounds on hands do not need to be kept away from food handling activities.**
1. True<br>2. False<br>3. Don’t know/Don’t remember
ATTITUDES
**1. Raw and cooked food should be stored separately.**
1. Disagree.<br>2. Don’t know/Don’t remember<br>3. Agree.
**2. The use of adornments, accessories or jewelry can contaminate food.**
1. Disagree.<br>2. Don’t know/Don’t remember<br>3. Agree.
**3. Wearing a cap is an important practice to reduce the risk of food contamination during handling.**
1. Disagree.<br>2. Don’t know/Don’t remember<br>3. Agree.
**4. Defrosted food must not be refrozen.**
1. Disagree.<br>2. Don’t know/Don’t remember<br>3. Agree.
**5. Eggs must be washed after purchase before being stored.**
1. Disagree.<br>2. Don’t know/Don’t remember<br>3. Agree.
**6. Food thawing can be performed in a bowl with or without water in the sink at room temperature.**
1. Disagree.<br>2. Don’t know/Don’t remember<br>3. Agree.

**7. Food must be cooled at room temperature before being put in the fridge.**
1. Disagree.<br>2. Don't know/Don't remember<br>3. Agree.
**8. Improper food storage may pose risk to health.**
1. Disagree.<br>2. Don't know/Don't remember<br>3. Agree.
**9. Preparing food in advance reduces the risk of contamination.**
1. Disagree.<br>2. Don't know/Don't remember<br>3. Agree.
**10. Using non-higienized fresh herbs in the decoration of a portion of broth or soup can contaminate these foods.**
1. Disagree.<br>2. Don't know/Don't remember<br>3. Agree.
**SELF-REPORTED PRACTICES**
**1. Do you wash your hands immediately before handling food?**
1. Never<br>2. Rarely<br>3. Sometimes<br>4. Most of the times<br>5. Always
**2. Do you use food after the expiration date if it has no change in quality aspect?**
1. Never<br>2. Rarely<br>3. Sometimes<br>4. Most of the times<br>5. Always
**3. Do you thaw food at room temperature (outside the fridge)?**
1. Never<br>2. Rarely<br>3. Sometimes<br>4. Most of the times<br>5. Always
**4. Do you check the expiration date of ingredients before using them in food preparation?**
1. Never<br>2. Rarely<br>3. Sometimes<br>4. Most of the times<br>5. Always

**5.**	**Do you wash your hands after using the bathroom?**
1. Never<br>2. Rarely<br>3. Sometimes<br>4. Most of the times<br>5. Always	
**6.**	**Do you talk while handling ready to eat food?**
1. Never<br>2. Rarely<br>3. Sometimes<br>4. Most of the times<br>5. Always	
**7.**	**Do you handle food when you are sick or have cuts on hands?**
1. Never<br>2. Rarely<br>3. Sometimes<br>4. Most of the times<br>5. Always	
**8.**	**Do you wear nail polish or use jewelry when handling food?**
1. Never<br>2. Rarely<br>3. Sometimes<br>4. Most of the times<br>5. Always	
**9.**	**Do you keep your hair completely covered with a cap while handling food?**
1. Never<br>2. Rarely<br>3. Sometimes<br>4. Most of the times<br>5. Always	
**10.**	**Do you sanitize your workplace after finishing your service?**
1. Never<br>2. Rarely<br>3. Sometimes<br>4. Most of the times<br>5. Always	

Final Score Instructions	
. For each correct item, assign one point to the final score.<br>. For incorrect items or 'do not know/do not remember', 'rarely' 'sometimes' and 'most of the times' answers, do not assign any points to the final score.	
**Classification**	
Food truck food handler with scores ≤ 6 in the knowledge section **OR** ≤ 5 in the attitudes section **OR** ≤ 6 in the self-reported practices section: high risk of contamination	
Food truck food handler with scores > 6 in the knowledge section **AND** > 5 in the attitudes section **AND** > 6 in the self-reported practices section: low risk of contamination	
